# Design and Evaluation of a Conductive-Knit Sensor System for Measuring Forearm Pronation and Supination

**DOI:** 10.7759/cureus.93323

**Published:** 2025-09-27

**Authors:** Masayuki Kajiura, Fumiaki Yano, Hiroya Fukuda

**Affiliations:** 1 Graduate School of Human Development and Environment, Kobe University, Kobe, JPN; 2 Textile Machinery Division, Control System and Development Department, Murata Machinery, Ltd., Kyoto, JPN

**Keywords:** conductive textiles, motion analysis, pronation, supination, textile sensor, wearable sensors

## Abstract

Wearable sensors enable continuous monitoring of human motion in daily life, but assessing forearm pronation and supination usually requires specialized laboratory equipment. This study aimed to design and evaluate a knitted textile sensor system based on carbon nanotube (CNT) conductive yarns for unobtrusive measurement of rotational forearm movements. To overcome the hysteresis inherent in knitted strain sensors, we implemented a mechanism using two sensors positioned at 45° on the forearm, arranged so that only one sensor undergoes elongation during each motion. This configuration leverages the sensor’s stable elongation response while minimizing the influence of unloading hysteresis, thereby enabling reliable estimation of rotational angles. Electrical characterization confirmed consistent strain resistance during elongation, and regression analysis showed that nonlinear (cubic) models provided superior accuracy, compared to linear models. Validation experiments demonstrated that the system achieved errors of about 2.5° root-mean-square in offline analysis and approximately 5° in real-time estimation, compared with a gyroscope. Importantly, the system did not require direct skin attachment and offered the advantages of breathability, flexibility, and comfort inherent to textile-based designs. These results highlight the feasibility of CNT-knitted sensors as a lightweight, cost-effective, and user-friendly platform for capturing complex upper limb movements, with promising applications in rehabilitation monitoring, sports performance assessment, and everyday healthcare technologies.

## Introduction

Wearable devices can help individuals conduct long-term, continuous health monitoring, tracking, and management. Such data can be particularly valuable when assessing the long-term efficacy of various clinical and medical interventions [1]. However, most of these devices are not suitable for complex issues, such as functional assessment of interventions for upper and lower limb rehabilitation, which requires specialized robotic systems [2]. It is therefore essential to develop methods that allow continuous monitoring and evaluation of limb movements in everyday settings.

Despite the advantages of this technology, several challenges prevent individuals from using and wearing these devices every day. Many complain that the devices are difficult or uncomfortable to wear. Breathability and flexibility thus are essential to ensure consistent and accurate measurements. Fiber-based sensors are flexible, lightweight, and breathable and thus well-suited for long-term monitoring [3-5]. Additionally, fiber-based sensors can be seamlessly integrated into garments, allowing for highly portable and unobtrusive use. Fiber sensors using carbon nanotubes (CNTs) have been proposed for use in collecting biological measurements due to their exceptional mechanical robustness and superior electrical and thermal conductivity [6-8]. In a comparison of CNT-based dry electrodes and conventional wet electrodes for electrocardiogram measurement, Hossain et al. confirmed the superior washability and long-term usability of CNT-based electrodes [9]. Doshi et al. proposed a pressure sensor capable of measuring pressures ranging from 10 kPa to 40 MPa by randomly orienting CNT fibers and coating them onto aramid nonwoven fabric that they placed in shoe insoles [10]. Another recent study explored the use of CNT-based fiber sensors for measuring lower limb gait [11]. Yet, research on their use in measuring upper limb movements and complex rotational motions such as pronation and supination remains scarce.

In this study, we aimed to design and develop a sensor system utilizing CNT-based knitted fabric to measure forearm pronation and supination. We also investigated whether the system can be used in daily life for real-time measurement of these types of movements. Specifically, we assessed (1) the electrical properties of the CNT-based knit fabric; (2) its ability to measure pronation and supination; and (3) the feasibility of using the system for real-time measurement. The findings indicate that CNT-knitted fabrics could be useful materials for use in sensors that capture forearm pronation and supination movements.

## Materials and methods

We conducted functional evaluations of the material and the sensor system, as well as real-time measurements. The material evaluation was conducted to support the development of the sensor system, the functionality of which was subsequently assessed via evaluation and real-time measurement.

Materials

**Figure 1:**
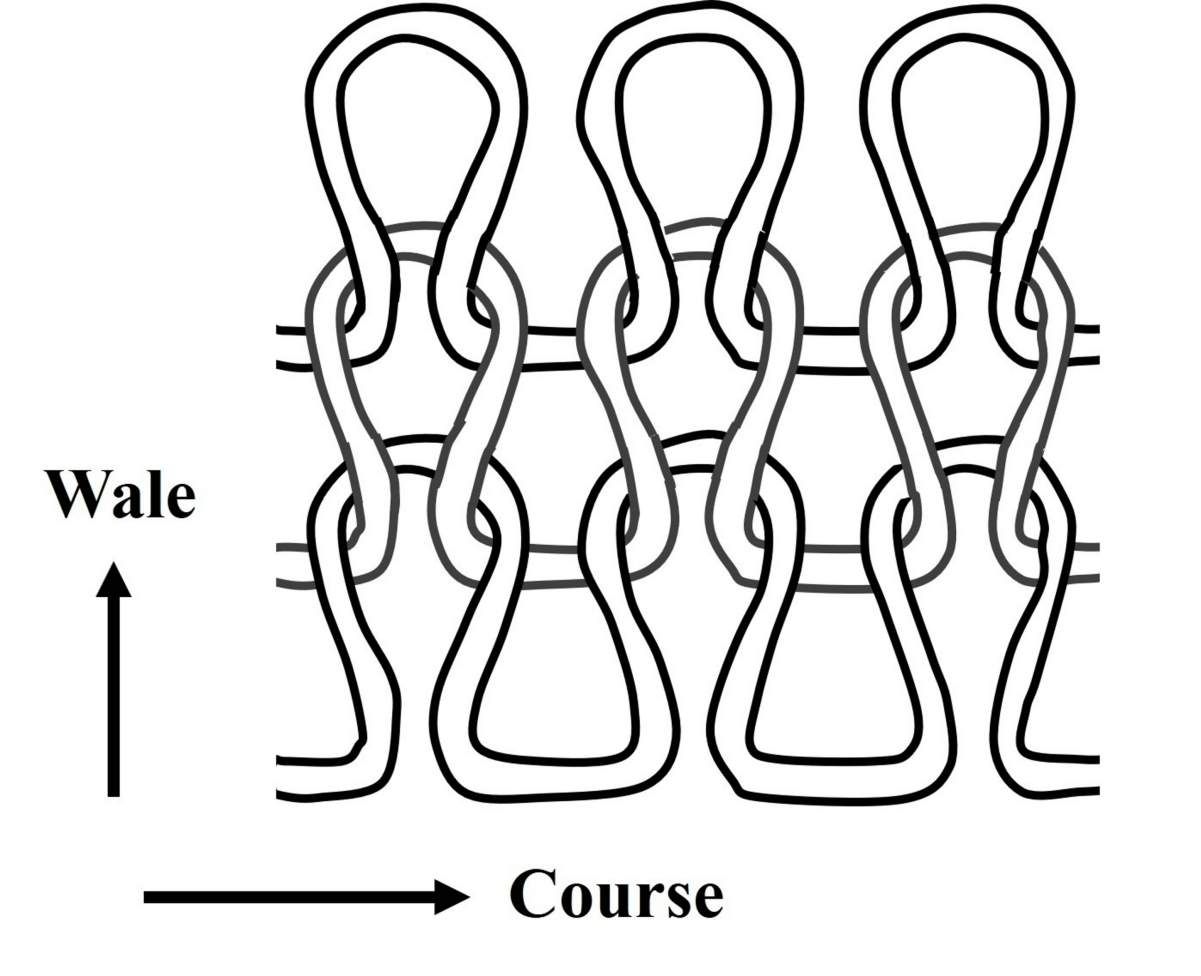
Structure of the course and wale configuration in the conductive yarn-knitted fabric Image Credit: Authors' original creation.

We designed a knitted fabric woven from conductive yarn composed of CNTs ($1 \, \Omega/\mathrm{m}$). In knitting terminology, horizontal rows of continuous loops are defined as the “course,” and vertical rows are defined as the “wale” (Figure 1). Knitting configurations are denoted by the number of courses × the number of wales. In this study, we used two knitting configurations (4 × 16 and 8 × 16), with initial lengths of 37 mm and 45 mm, respectively. Jansen reported that the resistance of conductive knits changes depending on the level of strain applied. Moreover, beyond a strain of approximately 20%, the resistance decreases due to reduced contact pressure between conductive yarns [12]. Accordingly, we pre-applied more than 20% strain in the course direction to offset the initial resistance variation.

Methods

**Figure 2:**
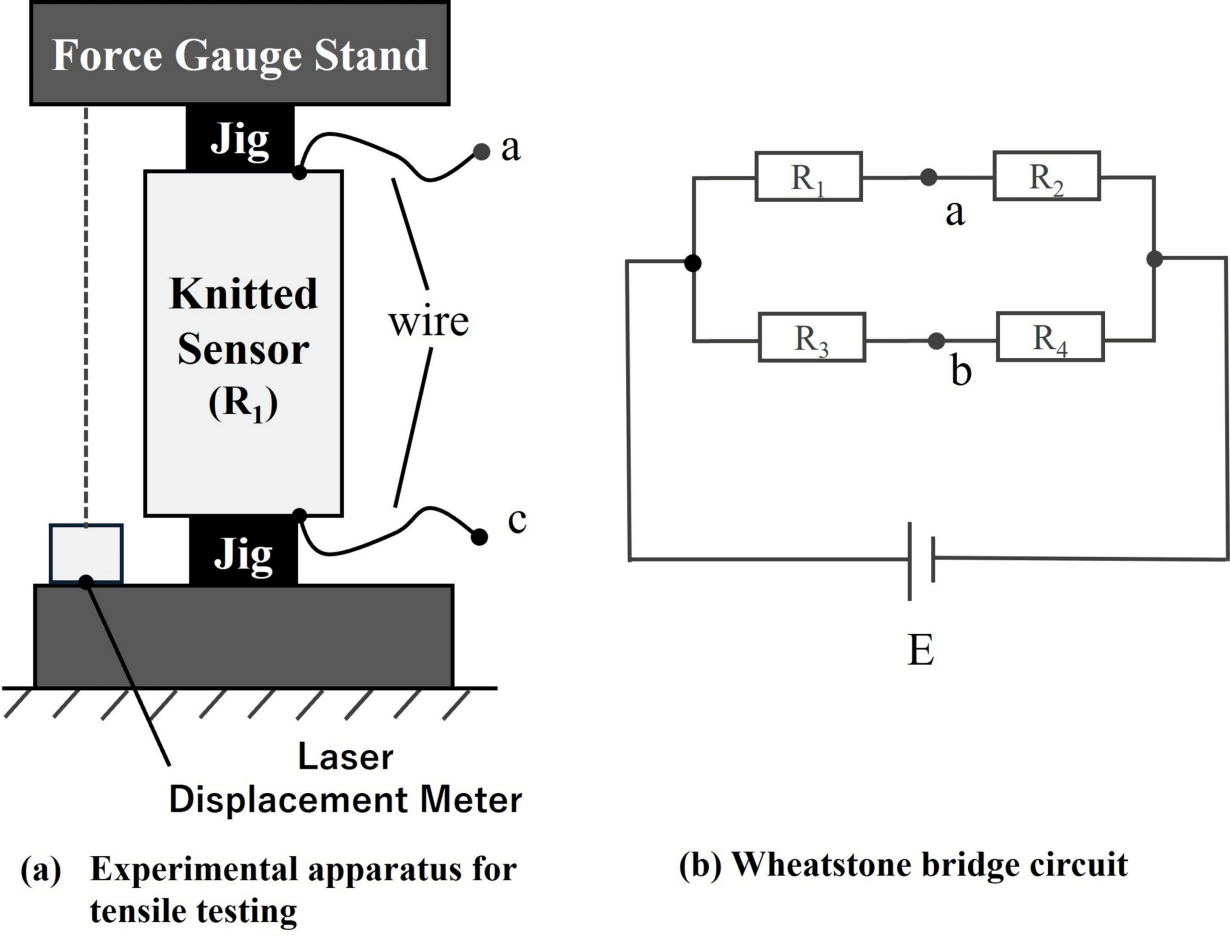
Experimental setup for evaluating the electrical characteristics of the knit sensor: (a) tensile testing apparatus and (b) the Wheatstone bridge circuit

To characterize the electrical properties of the conductive knit, we conducted tensile tests that enabled repeated measurements. As illustrated in Figure 2a, a force gauge stand (FGS-50E, Nidec Drive Technology Corp., Kyoto, Japan) was employed to elongate the knit at a constant speed (3 mm/s) and strain during loading and unloading and was measured using a laser displacement sensor (CD22, Optex FA Co., Ltd., Kyoto, Japan). Furthermore, as shown in Figure 2b, the electrical characteristics of the knit sensor were assessed using a bridge circuit, in which $V_a - V_b$ was determined as follows:

$V_a - V_b = \frac{R_2 R_3 - R_1 R_4}{(R_1 + R_2)(R_3 + R_4)} E$

Under the initial conditions, the knit sensor resistances were specified as $R_1 = r + \Delta R, \quad R_3 = r, \quad R_0 = R_2 = R_4$, with known resistor values. The resistance variation under the applied strain is given by the following expression:

$V_a - V_b = \frac{\Delta R {R_0}}{(R_0 + r + \Delta R)(R_0 + r)} E$

$\Delta R$ indicates the resistance change of the knit sensor resulting from mechanical strain, where $R_0 \gg r, \, \Delta R$,

$V_a - V_b = -\frac{\Delta R}{R_0} E$

From the above calculations, $V_a - V_b$ was obtained. Resistance variations in the knit sensor under the applied strain were recorded using a measurement system comprising a Wheatstone bridge and an instrumentation amplifier. These data were acquired via a microcontroller (Arduino Uno) at a sampling rate of 100 Hz.

The knit sensor was strained to approximately 40% of its initial length at a constant rate of 3 mm/s. Four loading-unloading cycles were performed, during which the electrical response of the sensor was continuously recorded. In addition, we observed changes in hysteresis, defined as the discrepancy in strain between loading and unloading at a given resistance change. To observe hysteresis changes that occurred along with the magnitude of strain, six elongation-unloading cycles were performed with varying strain levels. The resulting strain-resistance data were evaluated in terms of hysteresis and linearity and quantified using the following expression:

$H_\varepsilon = \frac{\Delta \varepsilon_{\mathrm{hys}}}{\varepsilon_{\mathrm{max}} - \varepsilon_{\mathrm{min}}}$

**Figure 3:**
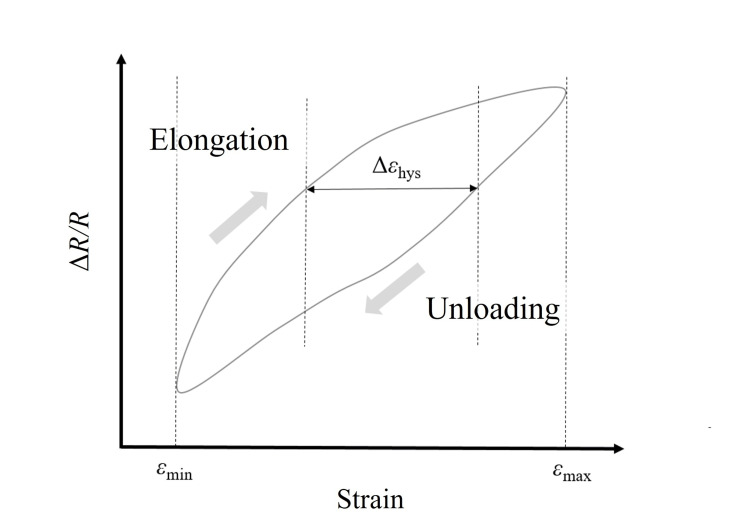
Hysteresis characteristics of the knit sensor during elongation and unloading cycles

As shown in Figure 3, $\Delta \varepsilon_{\mathrm{hys}}$ represents the maximum strain difference, with $\varepsilon_{\max}$ and $\varepsilon_{\min}$ denoting the maximum and minimum applied strain levels, respectively. For the linearity assessment, a linear regression model using the least squares method was employed for both the elongation and unloading phases. The root-mean-square error (RMSE) was calculated from the residuals [13].

**Figure 4:**
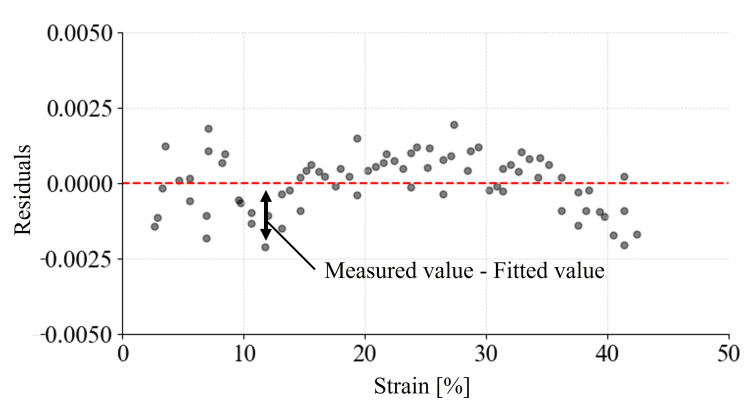
Residuals of resistance change in the knit sensor

Figure 4 presents the illustrative residual plot.

**Figure 5:**
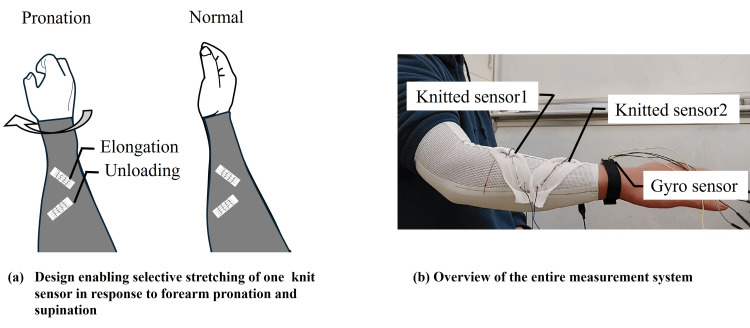
Measurement system for forearm pronation and supination utilizing knit sensors: (a) selective stretching on one sensor during movement and (b) movement of the entire measurement system

The knit sensors employed in this study exhibited hysteresis but maintained high linearity during elongation, consistently tracing the same resistance trajectory, irrespective of the elongation magnitude (see the Results section for details). To capture forearm pronation and supination, two knit sensors were integrated into an arm cover, as depicted in Figure 5a-5b. As shown, only one sensor underwent elongation during either motion. This deliberate design exploited the sensor’s consistent elongation behavior to reliably measure rotational forearm movements. To facilitate monotonic elongation with respect to motion, the sensors were pre-installed at a 45° angle relative to the forearm, thereby restricting elongation to a single directional axis.

The sensor system for measuring pronation and supination consisted of two previously described knit sensors (4 × 16 and 8 × 16), a measurement circuit integrating a bridge circuit with an instrumentation amplifier, a microcontroller, and a host computer. To capture rotational angles, a gyroscope sensor (BNO55, Bosch Corporation, Kanagawa, Japan) was employed. Pronation angles were designated as positive and supination angles as negative. Measurements were conducted with a single participant who performed five repetitions each of forearm pronation and supination, maintaining the forearm horizontally relative to the ground at four-second intervals. Changes in resistance from the knit sensors and rotational angles from the gyroscope were sampled at 100 Hz via a microcontroller. To evaluate the measurement system’s performance in capturing forearm rotational movements, regression models employing linear and cubic least squares fits were developed using the elongation outputs of the two knit sensors. Model accuracy was quantitatively compared via RMSE and root-mean-square percentage error. Model goodness-of-fit was assessed using the Akaike information criterion.

The proposed sensor system, which captures only elongation data even in sensors with hysteresis, is considered applicable to various motions such as ankle pronation/supination and knee flexion/extension. Real-time acquisition of pronation and supination angles is preferable in practical daily use. The knit sensor employed in this study exhibited a monotonic increase or decrease in resistance within the utilized strain range after a certain strain level was reached. Consequently, rotational direction related to pronation and supination could be determined by detecting whether the resistance change increased or decreased. We developed a real-time pronation/supination measurement system by applying numerical differentiation to identify rotation direction. We also collected real-time measurements to confirm its feasibility as a sensor system. With the same participant, we measured forearm rotation using the sensor system. The RMSE was subsequently calculated to evaluate the feasibility of the system.

## Results

Materials evaluation

**Figure 6:**
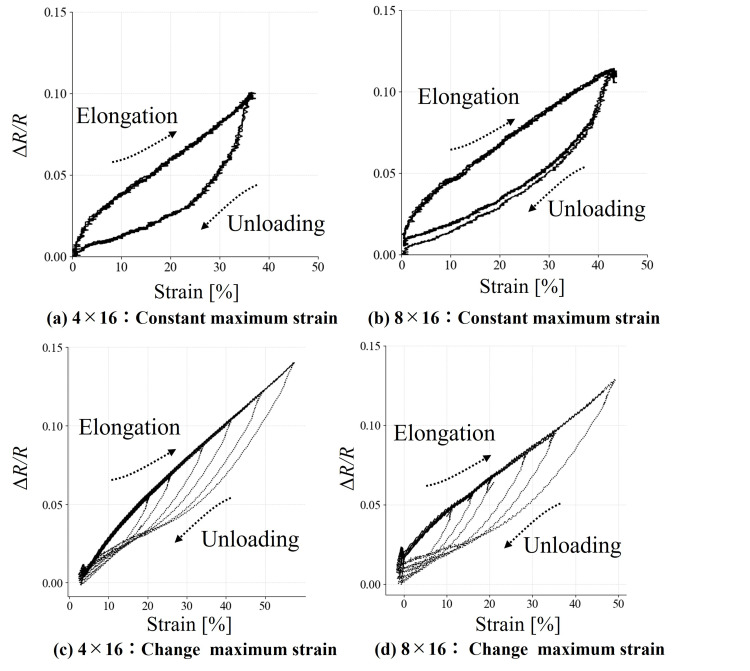
Relationship between strain and electrical property changes in the (a, c) 4 x 16 knit sensor and the (b, d) 8 x 16 knit sensor during loading and unloading

Figure 6a-6d reports the measurements for the knitted sensor. In each panel, the horizontal axis represents strain, and the vertical axis indicates the change ratio of resistance. As shown in Figure 6a, the knitted sensor exhibited hysteresis; however, during elongation, the RMSE was smaller, indicating higher linearity compared to unloading. In Figure 6b, the resistance change during unloading did not converge to a fixed value. Moreover, in Figure 6c-6d, different resistance changes were observed at the same strain during unloading. Table 1 presents the RMSE values for hysteresis and linearity. Overall, the results indicate that hysteresis occurred during unloading and that the knitted sensor exhibited stable electrical characteristics during elongation.

**Table 1:** Hysteresis under elongation and unloading, and linearity assessment via RMSE for the knit sensor RMSE: root-mean-square error.

Course × wale	Status	Hysteresis	RMSE (ΔR/R)	Coefficient of determination
4 × 16	Elongation	0.49	0.003	0.99
4 × 16	Unloading	0.49	0.011	0.85
8 × 16	Elongation	0.44	0.004	0.98
8 × 16	Unloading	0.44	0.008	0.92

Sensor system evaluation

**Figure 7:**
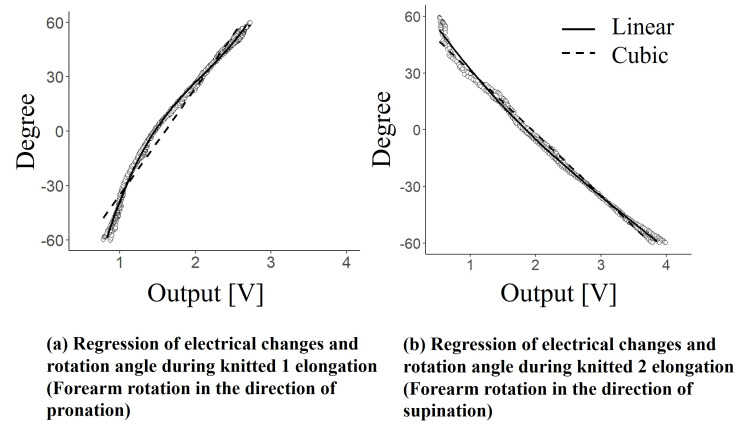
Results of regression of electrical changes and rotation angle during elongation: (a) pronation and (b) supination

Figure 7a-7b shows the relationship between the output of the knitted sensor and the rotation angle obtained during pronation and supination. For the accuracy evaluation of the approximation formulas for pronation, the coefficients of determination obtained using the linear and cubic models were 0.96 and 0.99, respectively. The Akaike information criterion values were 3535 for the linear model and 2915 for the cubic model, again showing superior fit for the cubic model. For both pronation and supination, the approximation using the cubic function demonstrated higher accuracy. As previously noted, the knit sensor’s electrical characteristics exhibited a nonlinear change with respect to strain, suggesting that the cubic model effectively captured this nonlinear relationship.

The RMSEs for the linear and cubic models applied to pronation motion were 6.7° and 2.5°, respectively. For supination, the RMSE values were 4.2° for the linear model and 2.5° for the cubic model. The cubic model consistently outperformed the linear model, yielding lower RMSEs and thus higher accuracy for both pronation and supination measurements. These results suggest that forearm rotational movements can be reliably quantified with an RMSE of around 2.5°, using cubic regression models.

Real-time evaluation

**Figure 8:**
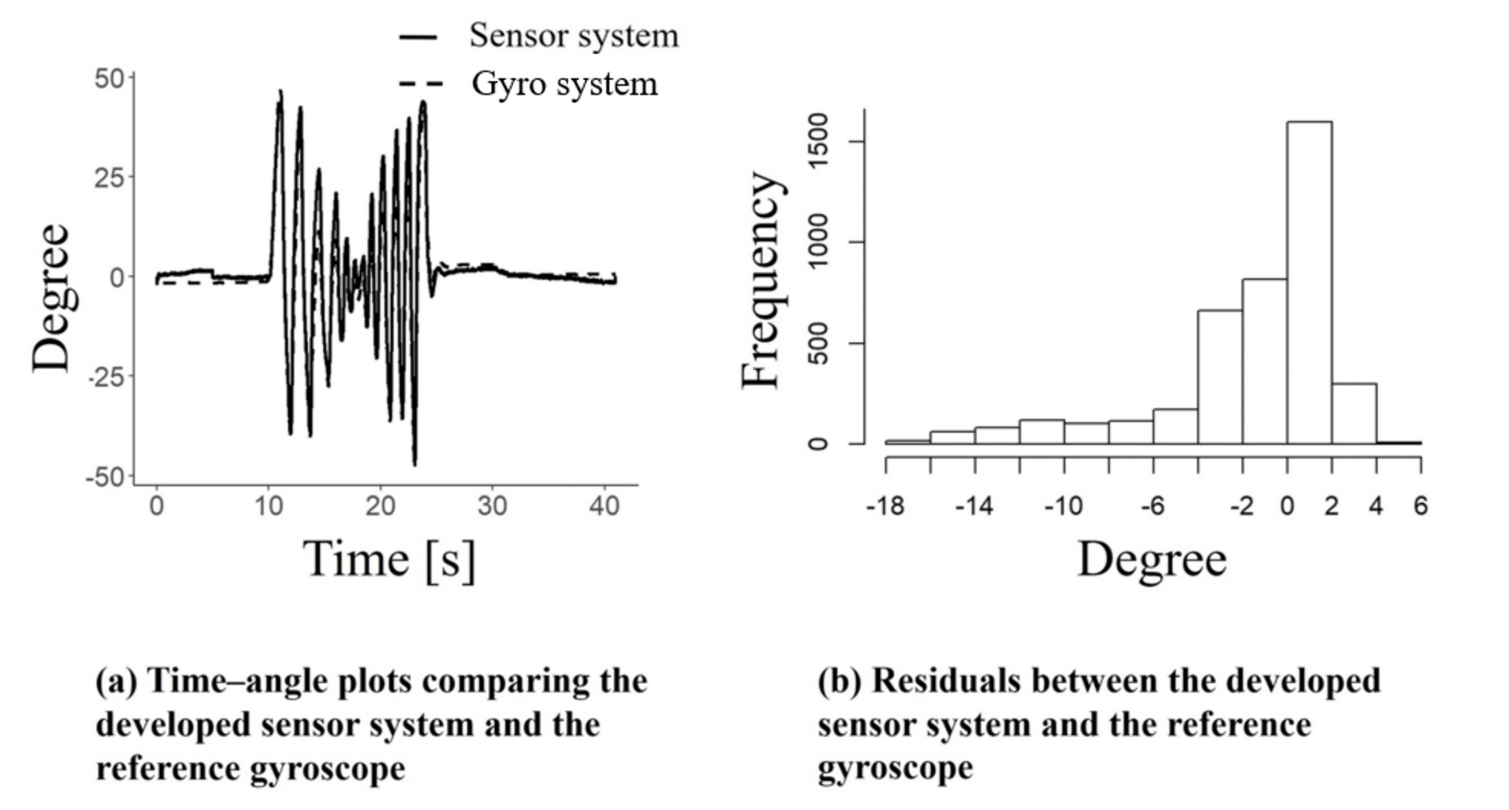
Real-time pronation and supination measurements from the knit sensor output: (a) waveforms and (b) residuals

Figure 8a-8b presents the measurement results of pronation and supination for one participant using the developed system in real time, along with residuals calculated from the gyro sensor data. In Figure 8a, the horizontal axis represents time, and the vertical axis indicates the pronation/supination angles obtained from both the gyro sensor and our sensor system. The RMSE was 4.3°. Figure 8b shows that most residuals fell within the range of -5° to +5°. These findings suggest that real-time motion measurement at a level comparable to prior research is feasible in daily life.

## Discussion

In this study, we designed, developed, and assessed a sensor capable of measuring forearm pronation and supination using a CNT-based knitted fabric. Our results show that despite the presence of hysteresis in the knit sensor, stable electrical responses during elongation permitted reliable measurements through a mechanism designed to elongate corresponding to pronation and supination. The sensor system achieved measurement accuracy with an RMSE of approximately 2.5° for both motions. Huang et al. reported forearm rotational measurements with an approximate 5° error by directly attaching dielectric elastomer-based strain sensors onto the skin [14]. The present work demonstrates equivalent rotational measurement accuracy without the need for direct skin attachment.

The knit sensor discussed herein exhibited hysteresis in its electrical response during elongation and relaxation cycles. Bozali et al. proposed a structure with reduced hysteresis by varying the number of knit courses and embedding conductive yarns on the interior, emphasizing that hysteresis is largely influenced by the properties of elastic yarns, rather than conductive yarns alone [15]. Warncke et al. demonstrated that knit patterns and sensor dimensions, such as length and width, significantly impact hysteresis, attributing its origin to residual strain [16]. Moreover, Shyr et al. identified that frictional interactions between conductive and elastic yarns, as well as fiber positional and morphological changes during mechanical deformation, contribute to hysteresis in strain sensors fabricated from conductive textiles [17]. Although low hysteresis can be attained by modifying knit architectures and elastic yarn materials, the extensive parameter space complicates the determination of an optimal configuration. This study shows that even with hysteresis, a knit sensor can achieve reliable correspondence with rotation angles by focusing solely on elongation-phase outputs, thus supporting the feasibility of using these sensors for such purposes.

In the measurements of pronation and supination, we found that the cubic regression model outperformed the linear model in accuracy. Specifically, the knit sensor exhibited a nonlinear relationship between strain and its electrical response. This nonlinearity was particularly evident as strain approached its peak, with measurement errors increasing alongside larger absolute rotational angles. Additionally, the knit sensor was affixed to the curved surface of the forearm. Given the anatomical curvature of the human arm, it is unlikely that the sensor’s elongation correlated linearly with the rotational angle. These considerations might explain why the cubic model provided a superior fit compared to the linear model. In addition, this study was conducted with a limited number of participants, and further validation with larger populations is required to confirm its applicability.

## Conclusions

In this study, we developed a sensor capable of measuring forearm pronation and supination using fabric knitted from conductive yarns. The sensor system demonstrated measurement accuracy with an RMSE of approximately 2.5° and real-time measurement with an RMSE of around 5°. Compared to traditional motion capture systems used for rehabilitation and upper limb motor function assessment, this sensor system could offer a cost-effective and user-friendly alternative. In future works, we aim to develop a system capable of simultaneously measuring forearm extension and pronation/supination, enabling comprehensive motion tracking in daily life. Given the small sample size, we also plan to validate the system with a larger participant group to ensure its generalizability.
