# Correction: Design and Evaluation of a Conductive-Knit Sensor System for Measuring Forearm Pronation and Supination

**DOI:** 10.7759/cureus.c400

**Published:** 2026-02-03

**Authors:** Masayuki Kajiura, Fumiaki Yano, Hiroya Fukuda

**Affiliations:** 1 Graduate School of Human Development and Environment, Kobe University, Kobe, JPN; 2 Textile Machinery Division, Control System and Development Department, Murata Machinery, Ltd., Kyoto, JPN

This article has been updated to correct the formatting for equations.

